# Deep learning-based image processing in optical microscopy

**DOI:** 10.1007/s12551-022-00949-3

**Published:** 2022-04-06

**Authors:** Sindhoora Kaniyala Melanthota, Dharshini Gopal, Shweta Chakrabarti, Anirudh Ameya Kashyap, Raghu Radhakrishnan, Nirmal Mazumder

**Affiliations:** 1 Department of Biophysics, Manipal School of Life Sciences, Manipal Academy of Higher Education, Manipal, Karnataka 576104 India; 2 Department of Bioinformatics, Manipal School of Life Sciences, Manipal Academy of Higher Education, Manipal, Karnataka 576104 India; 3 Computer Science and Engineering, Manipal Institute of Technology, Manipal Academy of Higher Education, Manipal, Karnataka 576104 India; 4 Department of Oral Pathology, Manipal College of Dental Sciences, Manipal, Manipal Academy of Higher Education, Manipal, 576104 India

**Keywords:** Optical microscopy, Image processing, Machine learning, Deep learning

## Abstract

Optical microscopy has emerged as a key driver of fundamental research since it provides the ability to probe into imperceptible structures in the biomedical world. For the detailed investigation of samples, a high-resolution image with enhanced contrast and minimal damage is preferred. To achieve this, an automated image analysis method is preferable over manual analysis in terms of both speed of acquisition and reduced error accumulation. In this regard, deep learning (DL)-based image processing can be highly beneficial. The review summarises and critiques the use of DL in image processing for the data collected using various optical microscopic techniques. In tandem with optical microscopy, DL has already found applications in various problems related to image classification and segmentation. It has also performed well in enhancing image resolution in smartphone-based microscopy, which in turn enablse crucial medical assistance in remote places.

**Graphical abstract:**

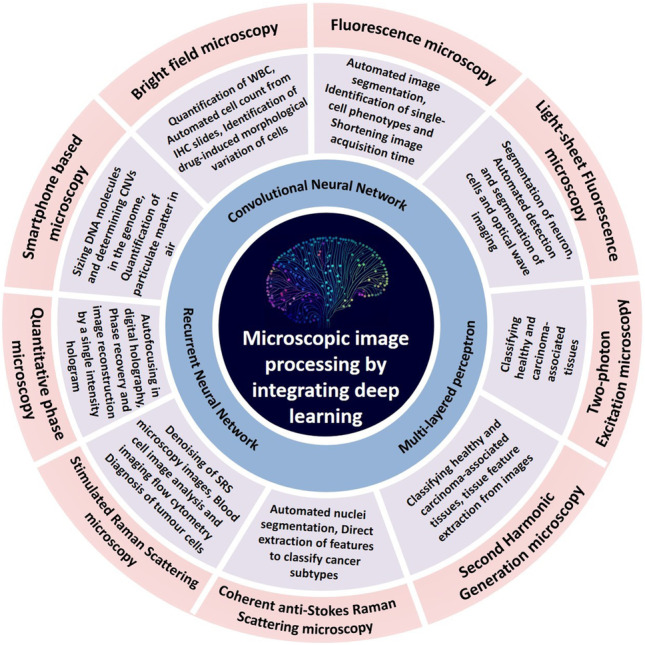

## Introduction

Biophotonics is an interdisciplinary field with flourishing applications in biomedical research, which allows for the delivery of clear insights regarding complex biological systems through the interaction of light with biological samples. However, modern optical imaging techniques that are label-free or employ exogenous labels such as wide-field microscopy, phase contrast microscopy, fluorescence microscopy, and nonlinear optical microscopy may be limited by the quality of output image, post image processing, or instrumentation cost (Mazumder et al. 2017). Manual image analysis of tissue samples is a very tedious and time-consuming process due to the complex nature of biological entities, which in turn demands an expert pathologist to record an accurate output. Besides, the manual analysis is highly subjective. To overcome these limitations, an automatic, fast, and robust image analysis technique that delivers well-processed images with defined image quality criteria is desirable (Rivenson et al. 2017).

**Fig. 1:**
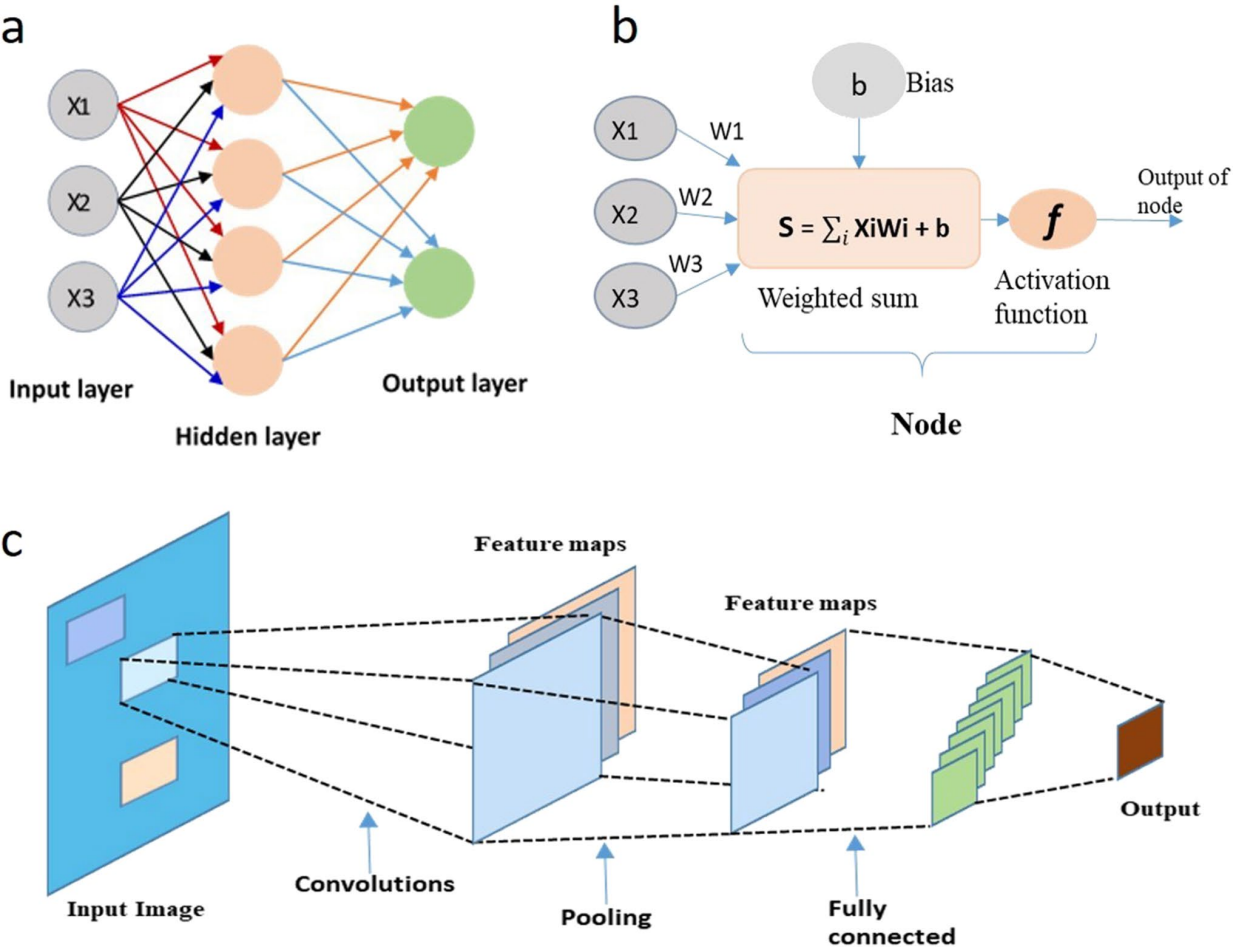
**a** Block diagram of a simple ANN architecture, **b** function of a node, **c** architecture of CNN in image classification of hepatocellular carcinoma graded into well, moderately, and poorly differentiated types (upper panel) along with image localization and segmentation. The image was reproduced with kind permission from Pradhan et al. 2020, Wiley

Machine learning (ML) is one such area of research, which performs statistical learning with the help of various multivariate analytical methods such as independent component analysis, principal component analysis (PCA), and multivariate regression. These methods aid in the identification of significant features during training of the ML algorithm, which may later be utilized for the classification or prediction of the test data set (Zhang, 2017). The ML algorithms such as support vector machine (SVM), artificial neural network (ANN), and cluster analysis are commonly implemented methods among which ANNs have proved to be very effective in addressing classification problems (LeCun et al. 2015). The ANN comprises a fundamental unit known as perceptron (Hornik et al. 1989) which is inspired by the biological neuron—an essential part of the biological neural networks. In general, any ANN consists of an input layer, a hidden layer, and an output layer (illustrated in Fig. 1). The input layer accommodates the neurons equal to the number of data points present in the data to be analyzed. The hidden layers may have a variable number of perceptrons optimized for the training set whereas the number of neurons in the output layer depends upon the problem addressed by ANN (Hornik et al. 1989). The input to every layer is drawn from the output of the preceding layer which is processed and translate to the next layer. The processing in each perceptron is performed in the form of an activation function as shown:

$${O}_{j}={\upsigma (\Sigma }_{i}{x}_{i}{.w}_{i}+{b}_{j})$$

Here, x_i_ is the input corresponding to the perceptron from the preceding layer, w_i_ are the parameters connecting different layers, b_j_ is the bias of the given perceptron and $\sigma$ represents an activation function. Simply, in a node, the weighted sum is calculated by adding b_j_ to the dot product of x_i_ with its corresponding w_i,_ and later the node is triggered based on the activation function $\sigma$ of the layer. Due to this process, ANN can perform as a universal functional approximator and hence used for analyzing data that address a variety of analytical problems (Hornik et al. 1989; LeCun et al. 2015; Kim et al. 2019; Rahman et al. 2018). Although ML algorithms perform well with a small amount of data, they are not fully automated and demand feature extraction from the input image. To overcome the limitations of ML techniques, ANNs with multiple layers, known as deep learning (DL) algorithms have been used (LeCun et al. 2015).

DL is a sub-category of ML, which provides improved performance with a prerequisite of advanced computing systems and an enormous amount of data to train the model. Although there is no specific thumb rule to calculate the size of dataset required for training, testing, and validating a model, DL algorithms require datasets in the order of thousands (Rivenson et al. 2017) whereas ML models require a few hundred (Kim et al. 2019) of data based on the complexity of the task performed. There are three different types of DL algorithms: multi-layered perceptron (MLP) with more than one hidden layer, convolutional neural network (CNN), and recurrent neural network (RNN). The MLP is a multi-layered ANN, in which the data is flown from input to output through hidden layers in the forward direction. CNN is a variant of ANN with learnable neurons which is specifically designed to analyze the spatial definition of the input dataset with more than one spatial dimension. In RNN, the feedback connections are used to store recent inputs given to the network to incorporate sequential data analysis (Pradhan et al. 2020; Elman, 1990; Deng and Liu, 2018; Wang et al. 2016). Among these techniques, CNN performs well with image processing and is widely used in biomedical image analysis to address a variety of problems such as image enhancement, classification, and segmentation. In general, the multi-dimensional dataset is fed to the CNN using the input layer. In the convolutional layer, randomly initialized kernels convolve with the input image to develop feature maps. Later, these feature maps are downsampled via a pooling layer using a pooling function. A CNN architecture may consist of multiple convolutional layers, which aids in significant feature extraction. Once the abstract features are extracted with convolution layers, the flattened features are fed into a fully connected layer for further classification (Pradhan et al. 2020; Nielsen, 2015). In the midst of wide-ranging applications of DL in data analysis, the functioning of these algorithms is not completely familiar to the researchers. However, by adjusting key variables known as hyperparameters, the performance of the same can be controlled during training (Hutter et al. 2019).

DL techniques are used to address a variety of imaging problems including, image resolution enhancement in ex vivo and in vivo imaging (Rivenson et al. 2017), image focus quality prediction (Yang et al. 2018), and diverse image set classification of different organelles (Huttunen et al. 2018; Kraus et al. 2017). In addition, the application of DL in mobile phone-based microscope has emerged as a potential tool for in analyzing biological specimens such as blood smear samples, tissue samples, and DNA (Rivenson et al. 2018b; Wei et al. 2014). It has also found application in cancer diagnosis to determine stages of cancer (Rehman et al. 2018; Esteva et al. 2017; Litjens et al. 2016; Chen et al. 2014), hyperspectral data classification (Malkiel et al. 2018), prediction of nanostructures of materials based on optical responses (Mohanty et al. 2016), and plant-based disease detection using mobile phone images (Li et al. 2019). Table 1 provides the brief summary of DL application in microscopic image analysis. In this review article, we discuss the applications of DL-based image processing in optical microscopy, which includes wide-field microscopy, fluorescence microscopy, light-sheet microscopy, second harmonic generation, coherent anti-Stokes Raman scattering (CARS), and stimulated Raman scattering (SRS) for improving the image quality, spatial resolution, and automatization.

**Table 1:** Applications of DL in different microscopic techniques

Types of microscopy	Principle and information	Application of DL	References
Bright-field microscopy	The specimen is magnified by the objective lens and transmits the light to the oracular lens or eyepiece	• Quantification of WBC • Automated cell count from IHC slides • Identification of highly accurate drug-induced morphological variation of cells	Jiao et al. 2019, Chen et al. 2014 & Kobayashi et al. 2017
Fluorescent microscopy	Refracted light passing through the objective is focussed on the fluorescent specimen	• Automated image segmentation/restoration • Identification of single-cell phenotypes and different cell types • Shortening image acquisition time and producing higher resolution images	Dumur et al. 2019, Krueger et al. 2019 & Wang et al. 2019
Light sheet fluorescence microscopy	Optical sectioning by illuminating the fluorescent sample with a thin sheet of laser light excites fluorophores	• Segmentation of neuron • Automated detection and segmentation of cells • Helps to study wave optical imaging in biological samples	Liu et al. 2017, Thierbach et al. 2018 & Weigert et al. 2019
Two-photon excitation microscopy	Photons combine their energy which allows low-energy infrared photons to excite fluorophores	• Classifying healthy and carcinoma-associated tissues	Huttunen et al. 2018
Second-harmonic generation microscopy	The non-linear coherent mechanism by which excitation of photons takes place from the lower energy state	• Classifying healthy and carcinoma-associated tissues • Tissue feature extraction from images	Huttunen et al. 2018
Coherent anti-Stokes Raman scattering microscopy	Displays the characteristic intrinsic vibrational contrast of the molecules	• Automated nuclei segmentation • Direct extraction of features to classify cancer subtypes	Huttunen et al. 2018
Stimulated Raman scattering Microscopy	The vibrational transition of the molecules due to the absorption of pump and Stokes photon	• Denoising of SRS microscopy images • Blood cell image analysis and imaging flow cytometry • Diagnosis of tumor cells	Manifold et al. 2019; Suzuki et al. 2019; Zhang et al. 2019
Quantitative phase microscopy	Quantifying of the phase shift when light travels through any optically dense specimen	• Automated identification of biological characteristics such as fingerprints • Autofocusing in digital holography • Phase recovery and image reconstruction by a single intensity hologram	Jo et al. 2017, Ren et al. 2018 & Rivenson et al. 2018a
Smartphone-based microscopy	The sample is placed directly on the surface of the image sensor	• Sizing DNA molecules and determining CNVs in the genome • Quantification of particulate matter in the air • Worm detection microscopic setup	Wei et al. 2014, Wu et al. 2017 & Bornhorst et al. 2019

## Applications of DL in labelled microscopy

### Bright-field microscopy

**Fig. 2:**
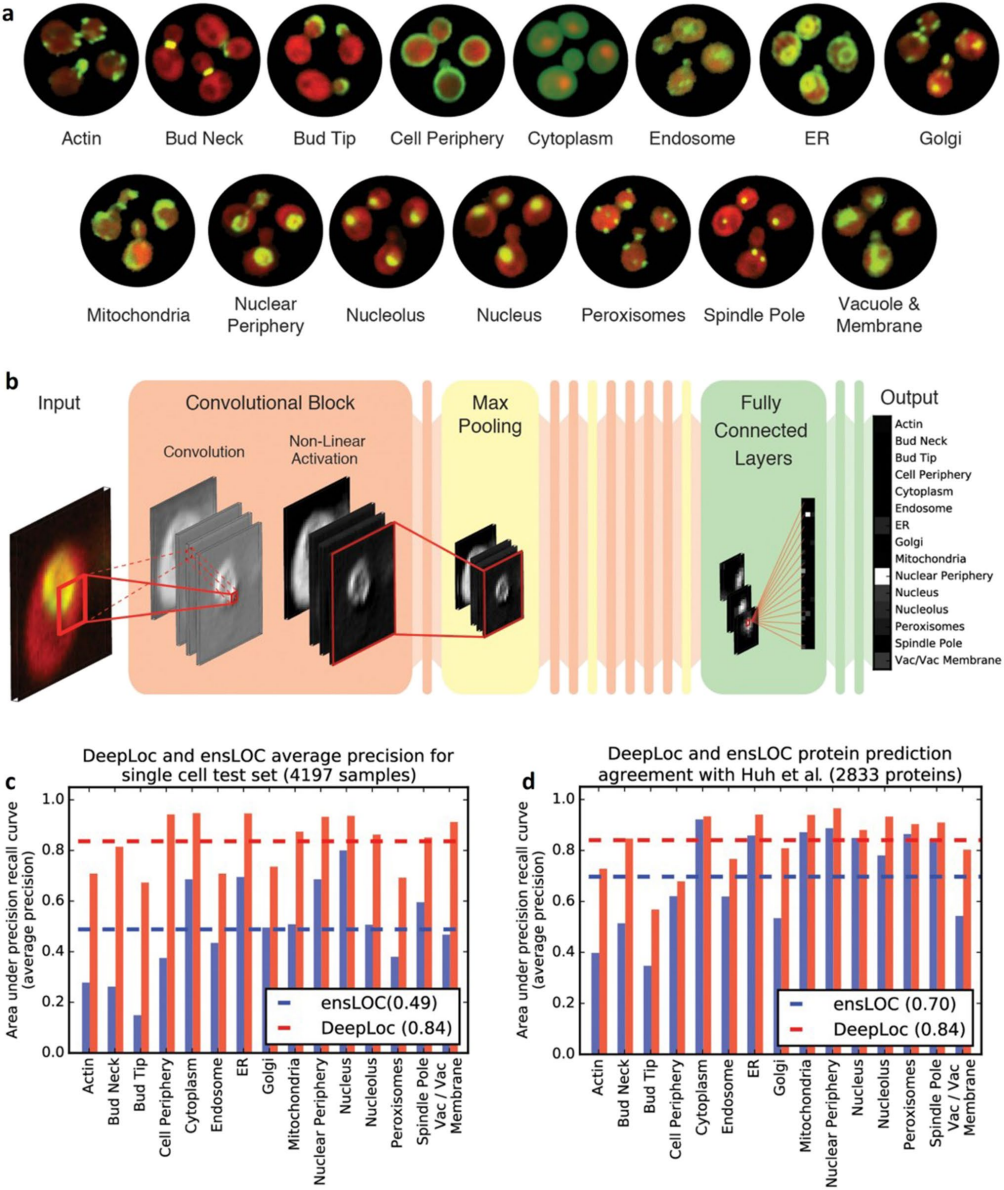
DeepLoc input data, architecture, and performance. **a** Example micrographs of yeast cells expressing GFP-tagged proteins that localize to the 15 subcellular compartments sed to train DeepLoc. **b** Architecture of DeepLoc. **c** Average precision of DeepLoc and ensLOC on classifying a single cell test set (*n* = 4197 samples). The dashed lines indicate the mean average precision across the localization classes (0.49 for ensLOC and 0.84 for DeepLoc). **d** Average precision of DeepLoc and ensLOC on assigning localizations to images of GFP fusion proteins with single or multiple localization classes (*n* = 2833 proteins). The dashed lines indicate the mean average precision across the localization classes (0.70 for ensLOC and 0.84 for DeepLoc). This figure is adapted with permission from Kraus et al. 2017, EMBO press

Quantitative analysis of microscopic data via existing computational programs counts on conventional machine learning techniques such as SVM, discriminative analysis, decision trees, and k-mean clustering (Hesamian et al. 2019). However, inadequate data selection during training, testing, and comprehensive examination of the model may lead to faulty results. Thus, a deep learning model undergone with rigorous training would help in overcoming the limitations associated with classical ML integrated image processing. A recent study revealed that the bright-field microscopic images of parasites in fecal samples can be automatically segmented and analyzed using a U-Net-based CNN which was initially trained to separate images of parasite eggs from the debris. This CNN was validated manually by a trained operator which confirmed that deep learning had improved accuracy over conventional image processing (Li et al. 2019). In another study, single-cell categorisation was employed by intelligent image-activated cell sorting (iIACS) technique integrated with DL-enabled real-time data analysis (Sozaki et al. 2019). For the study, DL-based software was installed for implementing the classifiers with the sort-decision function. CNN classification models were trained, validated, and tested using TensorFlow in the iIACS machine. Image augmentation was implemented to increase the training dataset, which provided additional variation in the input data. Further, the model parameters were tuned to achieve maximum classification accuracy through the validation dataset (Sozaki et al. 2019). A study conducted by Kraus et al. (as shown in Fig. 2) revealed that the use of deep CNN called DeepLoc enhanced the automated categorisation of protein subcellular localization in yeast cell images. DeepLoc outran the binary classifier ensemble (ensLOC) achieving an average precision of 84% and increasing the classification accuracy of the ensLOC by 15% across all localization categories. Further, transfer learning implemented on the fine-tuned model for the classification of various microscopic datasets showed promising results. Thus, DeepLoc could be used to analyze highly divergent microscopic images substantiating deep learning as one of the efficient approaches for the analysis of microscopic data (Kraus et al. 2017). In general, DL models outperform the classical ML models and transfer learning of a pre-trained DL model could save a lot of time and effort in collecting more data to train a new model.

Further, DL methods are also used in histopathological studies for automated cell counting, image segmentation and the monitoring of the drug response at a molecular level (Chen and Chefd’hotel 2014; Jiao et al. 2019; Kobayashi et al. 2017; Gallardo-Caballero et al. 2019). Chen et al. developed a cell count automation method on the digital images of immunohistochemistry (IHC) slides. The method included unmixing of colors to separate the IHC image into different color channels corresponding to cellular uptake. Deep learning was employed to formulate detection of the membrane image channel, as the biomarker of interest is membrane-bound. The algorithm was evaluated on a clinical data set demonstrating effective detection (Chen and Chefd’hotel 2014). In another study, Jiao et al. demonstrated a deep learning quantification and analysis system called DeepQuantify to analyze WBCs in light microscopic images of muscle tissue from female mice. This system segmented the images using the localized iterative Otsu’s thresholding method, masking post-processing, and classifying WBCs using a CNN classifier. Classification of WBCs was achieved with high accuracy with minimal manual intervention. The DeepQuantify system which employed a two-layered CNN architecture achieved better performance compared to other deep segmentation architectures such as FCN (fully conventional network), SegNet, and U-Net. It was fully automated, suitable for processing large image sets, and it could be used for quantifying various protein expressions (Jiao et al. 2019). Kobayashi et al. illustrated that combining bright-field microscopy with machine learning algorithms was useful in the identification of highly accurate drug-induced morphological variation in the cells (Kobayashi et al. 2017). The performance of this technique based on CNN architecture has yielded promising results in the pollen grain detection system (Gallardo-Caballero et al. 2019). Thus, the evolution of the DL algorithms has aided in the development of novel analytical techniques in histopathology which can outperform manual analysis. Although the training period for deep learning-based models is around 2–9 h, they provide accurate and reliable results. Notwithstanding this limitation, reducing the training period without compromising the quality of the image is the goal of DL.

### Fluorescence microscopy

Fluorescence microscopy entails the excitation and emission of labelled molecules present in the sample under study. It provides better contrast as compared to traditional optical microscopy due to the high specificity and selectivity of the fluorescent molecules present in the sample. A fluorophore absorbs a specific amount of energy, is excited to a higher energy state, and returns to the ground state emitting energy lower than the excitation energy. To process images captured by fluorescent microscopy, DL algorithms have been developed. Function-specific tasks in bioimaging employed by DL algorithms such as Cell Profiler 3.0 enable automation of image segmentation/restoration (Dumur et al. 2019). Additionally, DL algorithms such as ILASTIK and cell classifier can be used for 2D/3D image classification, segmentation, and analysis of fluorescent microscopy images. It was suggested that intelligent image processing was beneficial for the analysis of complex 3D/4D interactive images compared to supervised learning. This method ensured the delivery of the 3D view without compromising spatial dynamics (Dumur et al. 2019). Yao et al. developed and validated a deep CNN named Net-FLICS (fluorescence lifetime imaging with compressed sensing) that was used to record fluorescence intensity and lifetime imaging from single-pixel datasets, enabling the generation of quantitative intensity maps and fluorescence lifetime images (FLI), yielding superior results at low photon count (Yao et al. 2019).

Prediction of 3D fluorescence directly from the images of transmitted light was proposed by Ounkomol et al. They presented a tool based on U-Net architecture for modelling relationships between diverse but correlated imaging modalities, and its effectiveness in predicting fluorescence images (Ounkomol et al. 2018). Another recent study suggested the application of CNN to enhance the fluorescein sodium-driven intraoperative confocal laser endomicroscopic imaging model, where image style transfer modified the pixel values of the target image while the model parameters were fixed (Izadyyazdanabadi et al. 2019). Krueger et al. developed a scalable visual analytics application termed Facetto to discover single-cell phenotypes in high-dimensional multi-channel microscopy images of human tumours and tissues combining unsupervised and supervised learning for hierarchial phenotype analysis (Krueger et al. 2019). For image segmentation, Caicedo et al. presented an evaluation framework to measure accuracy, types of errors, and effectiveness of DeepCell and U-Net. Using neural networks, models were trained with small datasets, making them usable in the small data regime for image segmentation. This framework revealed that DL can reduce a significant number of biologically relevant errors (Caicedo et al. 2019). A novel DL-guided Bayesian inference (DLBI) approach was proposed for the time-series analysis of fluorescent images combining DL and statistical inference factoring three main components: (a) a simulator that provides the high-resolution input image by simulating low-resolution time-series fluorescence images to create supervised training data, (b) a deep learning module to capture spatial information in the low-resolution image and temporal information among the time-series images, and (c) Bayesian inference module that took the image from the deep learning module and removed artefacts by statistical inference, and therefore providing more accurate results (Li et al. 2018).

**Fig. 3:**
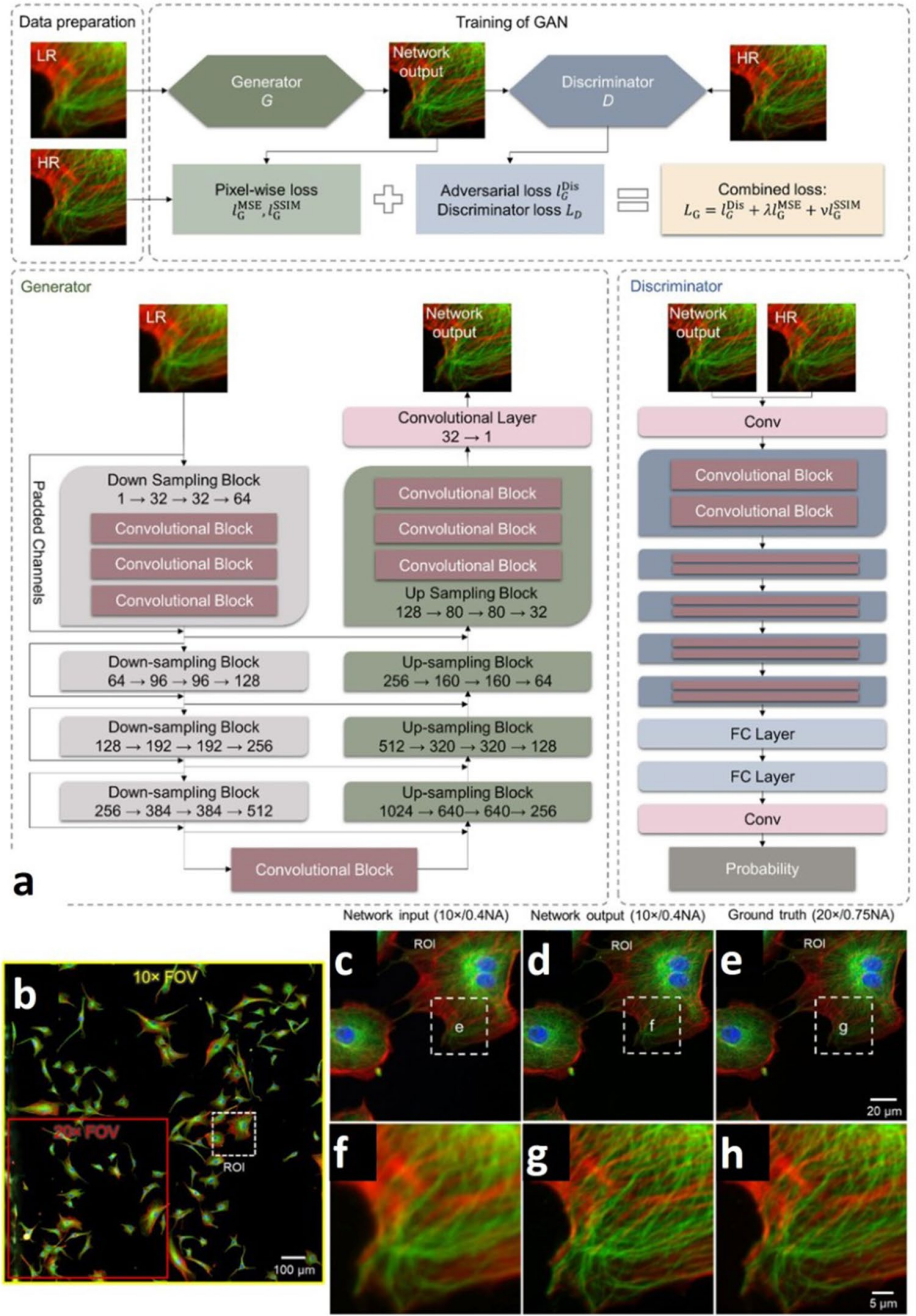
**a** The training process and the architecture of the generative adversarial network that we used for super-resolution of images of bovine pulmonary artery endothelial cells (BPAEC). **b** Network input image acquired with a 10 × /0.4NA objective lens. A small ROI is zoomed-in and shown in **c** network input, **d** network output, **e** ground truth (20 × /0.75NA). **f**–**h** Further zoom-in on a cell’s F-actin and microtubules. This figure is adapted with permission from Wang et al. 2019, Springer Nature

Super-resolution fluorescence microscopy has become an essential tool for the direct visualization of biomolecules at nanoscale resolution. Existing methods have limitations which include a longer time of execution, artificial thinning-thickening of structures, and lack of capacity to capture latent structures. Fluorescence microscopic data when combined with CNN analysis for image restoration, deconvolution, super-resolution, virtual staining, image segmentation, classification, and phenotyping has demonstrated major benefits (Belthangady and Royer, 2019). Another study showed that content-aware image restoration (CARE) helps in the improvement of acquisition parameters, such as photo-toxicity, speed of imaging, isotropy, or resolution. Therefore, fluorescence microscopes operated at shorter exposures with higher frame-rates, and lower light intensities in combination with content-aware restorations, approaches with higher resolution, and enhanced downstream analysis (Weigert et al. 2019). A DL-based framework was presented to obtain super-resolution using a generative adversarial network (GAN) model in fluorescence microscopy. In the study, the images of pulmonary artery endothelial cells acquired with the10 × /0.75NA objective were compared with the images taken with the 20 × /0.75NA objective during GAN training. This converts diffraction-limited input images into super-resolved images offering a larger field of view (FOV), and depth of field (DOF) allowing a short image acquisition time, and freedom for imaging vulnerable objects (as shown in Fig. 3) (Wang et al. 2019; Pinkard et al. 2019). Another study proposed that Deep-Z digitally increases the DOF without axial scanning or compromise in imaging resolution. It improved volumetric imaging speed, signal-to-noise ratio, and generated a virtual image that is temporally synchronized through digital refocusing. Additionally, it had the potential to reduce the photobleaching of samples associated with volumetric imaging (Wu et al. 2019a, b). Different fluorescent dyes and light sources were used in this microscopic technique which made the noise elimination essential for analysis. Pre-trained DL algorithms helped in eliminating noise and producing super-resolved images, thereby making it possible to directly extract useful information. Unsubstantiated details may be misleading, thereby making label-free detection in addition to unknown structure discovery and nanoscale image resolution a focus of future research.

Further, in a study conducted by Liu et al. the drawbacks with fluorescence imaging, owing to diffraction and noise are added separately with DL algorithms and alternating directions method of multipliers respectively. The decoupling of the subproblems high generalization with fidelity and enhanced resolution (Liu et al. 2020). In another study, 5 autophagy (ATG)-related genes of *S. cerevisiae* were studied with respect to time by inducing autophagy using nitrogen starvation. A new DL tool called DeepPhagy was designed for recognizing autophagic cells with superior accuracy. This tool was used to analyze the obtained images and classify the phenotypes quantitatively by measuring the autophagic activity. High consistency was observed in the classification, thereby suggesting that DL-based methods could be applied to study different types of autophagy (Zhang et al. 2020a, b, c).

### Smartphone-based microscopy

Smartphone-based microscopy is one of the emerging technologies in biophotonics with many futuristic applications. They are portable and cost-effective in comparison with laboratory-grade microscopic techniques. However, the performance of smartphone-based microscopes is limited by the sensory distortions in imaging microscopic specimens such as geometrical shrinkage, aberrations with higher numerical aperture lenses, etc. In this regard, the application of DL algorithms in smartphone-based microscopy can enhance the resolution of the image. Rivenson et al. reported the use of DL to correct such distortions, resulting in high-resolution, denoised, and color-corrected images, achieving a performance close to high-end optical microscopes. The experimental setup consisted of Nokia Lumia 1020 mobile phone equipped with additional optical components such as laser diodes and focusing knobs for imaging along with a series of 12 RGB LEDs as a light source to illuminate the sample. The samples such as lung tissue, blood smear, and Papanicolaou were imaged on the 3D printed optomechanical stage. While training the algorithm, bright-field microscopic images acquired with a 100 × objective lens were considered as reference images and were compared with the images acquired with smartphone setup. After training and validation, the algorithm was tested with fresh sample images to score the performance (Rivenson et al. 2018b; Kraus et al. 2017). Additionally, UCLA Samueli School of Engineering Researchers demonstrated that deep learning can enhance microscopic details in smartphone images and improve the resolution and color to approach laboratory-grade microscope performance (Rivenson et al. 2018b).

**Fig. 4:**
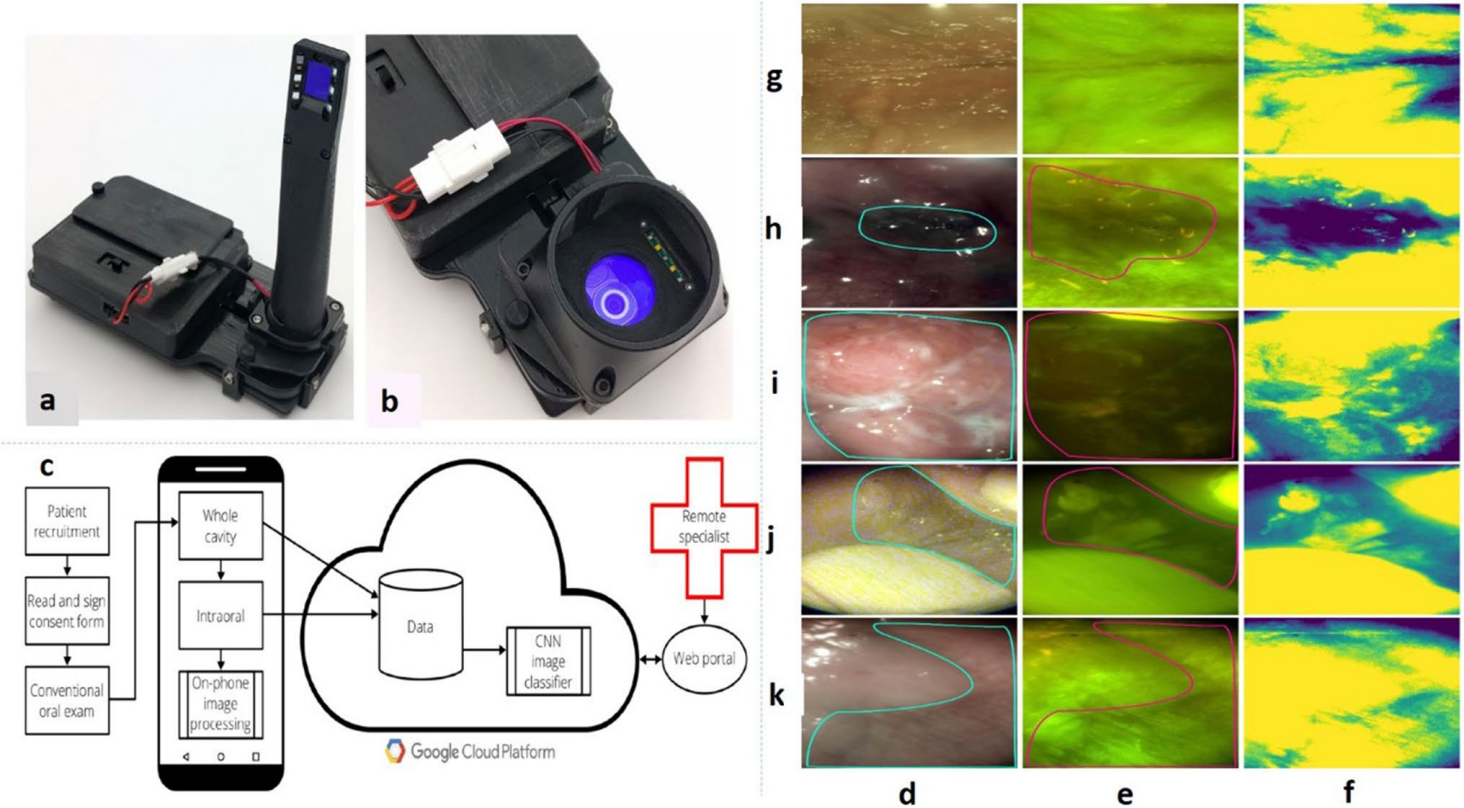
Smartphone-based oral cancer screening device using both WLI and AFI. **a** Intraoral imaging device. **b** Whole cavity imaging device. **c** Field testing workflow for smartphone-based oral screening. **d** White light imaging. **e** Autofluorescence imaging. **f** Green intensity map with the mean subtraction. Whole cavity module field testing images with suspect areas outlined. On-site specialist diagnoses were **g** normal/variation, **h** homogeneous leukoplakia, **i** carcinoma of the left mandibular alveolus, **j** tobacco pouch keratosis demonstrating increased fluorescence due to hyperkeratosis, and **k** tobacco pouch keratosis. This figure is adapted with permission from Uthoff et al. 2018, PLOS

Wei et al. reported a solution for sizing DNA molecules and determining copy number variations (CNVs) in the genome using a field-portable and cost-effective design for detection of nervous system disorders, cancers, or drug resistance utilizing the mobile phone-based imaging platform (Wei et al. 2014). Uthoff et al. developed a device LenCheck with an intraoral imaging facility to achieve a quicker diagnosis of oral cancer in remote areas. An LG Android smartphone was used in the experiment, which was capable of imaging the whole oral cavity along with intraoral imaging (as shown in Fig. 4). Both auto-fluorescence and bright-field images could be acquired with the developed device, where auto-fluorescence enabled early detection of cancer. The LensCheck system measured the point-spread function (PSF) of the intraoral lens system without the smartphone camera lens or image sensor and the modulation transfer function was determined by the normalization of the Fourier transform of the PSF. Positive predictive value (PPV), negative predictive value (NPV), sensitivity, and specificity were determined to compare the CNN result with gold-standard remote specialist diagnosis (Uthoff et al. 2018).

In comparison to the above, smartphones have also been employed with different machine learning techniques to perform a variety of tasks. A study was conducted to quantify, particulate matter (PM) in the air around Los Angeles International Airport (LAX), USA, using a device called c_Air which could screen 6.5 L of air in 30 s. Custom-developed machine learning algorithms were employed for the detection and sizing of different particles in air (Wu et al. 2017). In addition to this, a group of researchers developed a microscopic smartphone-based worm detection setup incorporating a support vector machine (SVM) for the detection of *Caenorhabditis elegans* (*C. elegans*). A Samsung S7 smartphone with additional lenses was used to image the cultured wild-type *C. elegans* placed on a petri dish. The SVM algorithm was trained with histogram orientation gradient (HOG) features extracted from 240 images accumulated using the setup. The technique achieved remarkable accuracy with 90% sensitivity and 85% specificity (Bornhorst et al. 2019). Mobile phone-based microscopy was developed with a focus to create low-cost and portable devices.

### Light sheet fluorescence microscopy

Light-sheet fluorescent microscopy (LSFM) is a type of fluorescence microscopy having moderate to high resolution and high speed. LSFM performs rapid 3D volumetric imaging of living cells with low phototoxicity. However, LSFM is limited in the axial resolution of ~ 4 to 20 µm which is not enough for 3D visualization of neurons. Liu et al. presented 3D isotropic LSFM imaging of the mouse brain in combination with multi-view imaging computation. The brain was visualized three-dimensionally by using under eight FOVs using a homemade selective plane illumination microscopy (SPIM). The resultant images provided the structural information and a resolution beyond the FOV of the microscope. It was further studied with the help of Bayesian-based multi-view deconvolution (MVD) and registration for complete details with proper segmentation of a single neuron. MVD-LSFM was demonstrated as a cost-effective, simple arrangement with better performance. The study showed good potential for the development of histopathology, neuroscience, and tissue or organ research (Liu et al. 2017).

Silvestri et al. showed that optical microscopy, when coupled with other advances could improve resolution and imaging quality. In this study, the distribution of Purkinje cells (PCs) in the entire cerebellum of the L7-GFP transgenic mouse was characterized. These PCs were fluorescently labelled, and an algorithm was used for the identification and localizing of the same. The Purkinje layers containing no cells were recognized using a vectorized representation of the cell population. This approach was used to study the distribution of various cells throughout the brain. But it led to the limitation of decreased image volume with the increase in resolution. Therefore, software tools used could handle large data for both processing and storage (Silvestri et al. 2015). DL integrated image processing improves the accuracy of the data; however, concepts of topology or smoothness could be hard for ANN to encode. Thierbach et al. presented DL-based light-sheet microscopy and geometric models preserving topology. In the study, active contour methods which are widely used in computer vision to perform image segmentation were used. The counters are boundaries designed for the area of interest in the image and are found to provide smoothness and uniform topology. However, these methods required an approximate number and position of objects in the image. In this study, the strengths of CNNs and active contour methods were combined to obtain good quality results by detecting and segmenting cells with the least annotations. For further improvement in cell detection, future research may focus on the optimization of the regression network for better detection of cells (Thierbach et al. 2018). Hay et al. demonstrated the use of CNN-based 3D light-sheet fluorescence microscopy imaging of larval zebrafish intestines. It achieved high accuracy and operated rapidly with two-fold improvements compared to other standard methods of machine learning. It was suggested that accuracy depended on dataset size and augmentation (Hay and Parthasarathy, 2018).

**Fig. 5:**
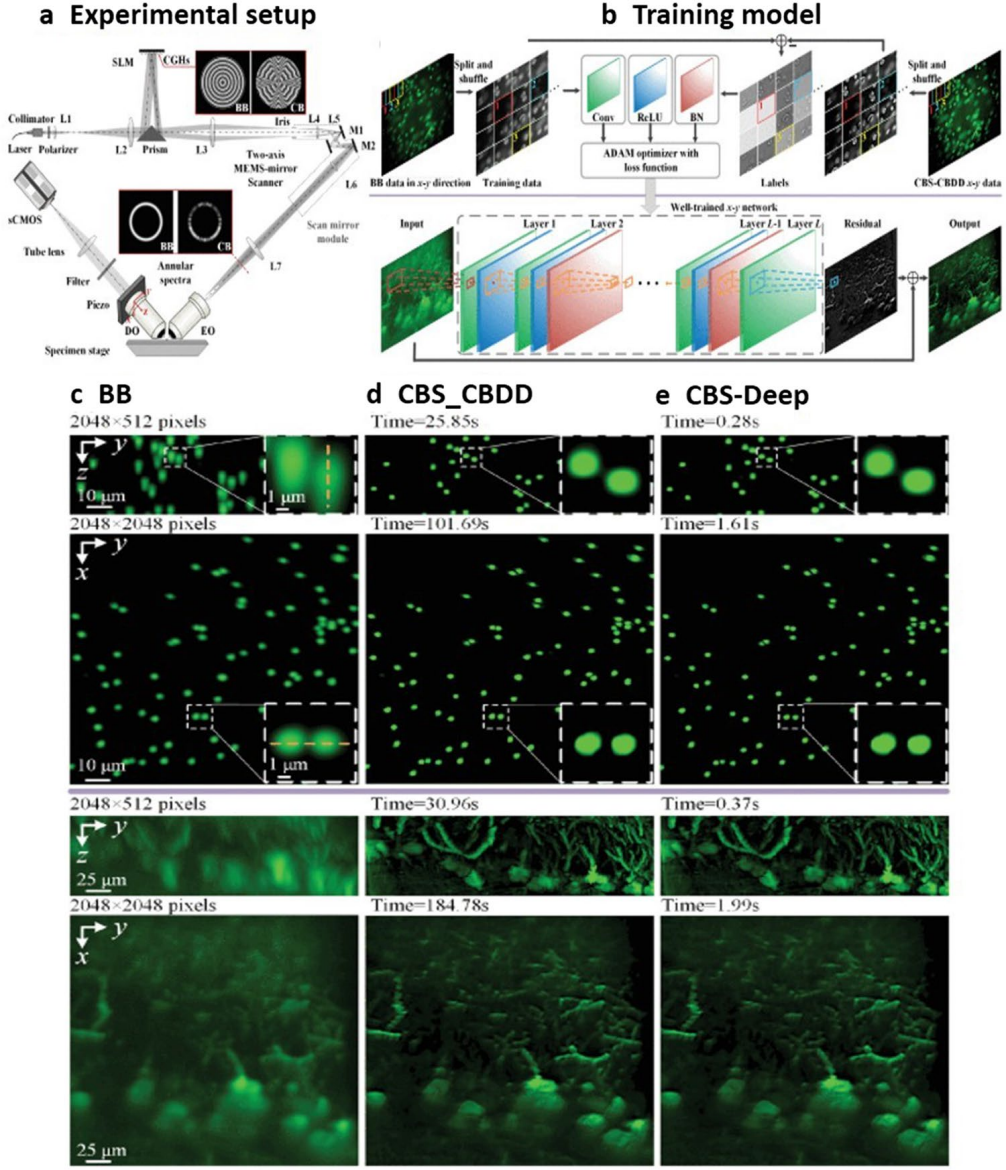
**a** Schematics of the CBS-based LSFM imaging system. **b** Training of the network using a BB image and a corresponding CBS-CBDD reference image in the *x*–*y* direction and then reconstructing the high-quality output image using this trained *x*–*y* network. Comparison of the testing images, i.e., first row for fluorescent microspheres and the second for mouse brain section, obtained by **c** BB, **d** CBS-CBDD, and **e** the CBS-Deep methods, respectively. This figure is adapted with permission from Bai et al. 2019, IEEE Photonics Society

Rieker et al. proposed a cost-effective and flexible light-sheet microscopy for rapid 3D imaging of protein dynamics in model organisms and tissue samples. This system enabled multi-fluorescent imaging with low photobleaching and was capable of monitoring protein localization and gene expressions. Fluorescence recovery after photobleaching (FRAP) assays were performed in cells and tissues of *C. elegans*. Future work may focus on the combination of microfluidics platforms to perform this study on the higher organisms (Rieckher et al. 2015). The complementary beam subtraction (CBS) method using non-diffracting as well as self-reconstructing Bessel beam (BB) and double scanning could decrease the background signal due to out-of-focus and axial resolution. The blurring and noise experienced during CBS imaging could be avoided with the help of a compressed blind deconvolution and denoising (CBDD) algorithm. This method however needs double scanning which results in huge calculation costs. A deep learning tool for Bessel beam based LSFM, capable of recreating good quality images is depicted in Fig. 5. As compared to the CBS284 CBDD method, the quality of image attainable with this CBS-Deep method was found to be competitive, whereas the speed of image reconstruction was 100 times faster. Increased feasibility was achieved by decreasing reconstruction time and improving the scanning practices. This cost-effective and suitable method resulted in good quality images using the light sheet imaging technique. DL-based light-sheet fluorescence microscopy exhibits robust cell segmentation, improved cell detection and, very accurate microscopy data along with faster performance (Bai et al. 2019).

Light scattering is one of the major challenges in live imaging applications. Xiao et al. reported a DL approach ScatNet that reverses the high-resolution 3D fluorescence microscopy targets to less quality and light-scattered measurements. This restored the light-scattered and blurred images of deep tissue. ScatNet approach can improve the imaging depth and its combination with LSFM was used in the restoration of cell nuclei images from the live *Drosophila melanogaster* embryos. It was also be applied with two-photon excitation microscopy for mouse brain imaging leading to improvement in the SNR and neuron resolution. It could help in widespread applications in image segmentation, and imaging away from the superficial cortex area (Xiao et al. 2020). Recovering the lost resolution by selective sub-sampling to obtain a wider field of view in a proper time scale was demonstrated by Corsetti et al. by combining a DL super-resolution with light-sheet microscopy by airy beam to obtain high-resolution images than better FOV and DOF. This method was implemented in the imaging of amyloid plaques in the mouse brain with Alzheimer’s disease, tissues of breast and colon cancer, and healthy tissues (Corsetti et al. 2020). LSFM, fast-volumetric imaging has been highly prone to artefacts along with being highly effective throughput. A novel framework of hybrid light-sheet microscope and DL-based volume reconstruction in which the single light-sheet provides validation and training data for CNN generating the volumetric microscopic data. The framework was applied for the heart dynamics of medaka and the neural activity of zebrafish. It was found to achieve good quality image reconstructions at high throughput (Wagner et al. 2021).

## Application of DL in label-free microscopy

### Quantitative phase microscopy

Quantitative phase imaging (QPI) is a label-free imaging technique that generates a quantitative image of phase delay in the optical path through the specimen. It is a cost-effective, less time-consuming technique that also eliminates the requirement for chemical reagents for staining. Rivenson et al. reported that DL-based holographic image reconstruction methods were found to be robust and rapid. In the study, CNN was trained using high-quality image labels which provided a high reconstruction speed in comparison with the iterative algorithms (Rivenson et al. 2019b). PhaseStain, a digital staining method has been developed which allows the transformation of quantitative phase images into bright-field microscopy-like images of the same histologically stained sample. This was performed by training a GAN (Rivenson et al. 2019a). Jo et al. proposed a method for screening *Bacillus anthracis* using DL-based holographic microscopy. In this method, a deep CNN was trained with unlabelled holographic images of living cells to achieve high subgenus specificity and single-spore sensitivity. A CNN named HoloConvNet was designed for the holographic image classification of each cell. This “Representation learning” method enables the training of raw images directly instead of using features, extracted manually. Biologic characteristics are identified and utilized as fingerprints. Intelligent holographic microscopy could identify and utilize classified fingerprints. It is believed that this strategy will result in the accessibility of holographic microscopy to doctors and scientists to perform rapid and accurate diagnoses leading to novel applications (Jo et al. 2017).

Digital holographic microscopy (DHM) is used to create a hologram of the sample by encoding a 3D optical field into intensity modulations through the interference of reference and scattered waves of the sample. However, challenges of DHM include missing phase, the appearance of coherence-related artefacts like speckle noise and reflection interference reducing the contrast of the image thereby affecting its utility. Ren et al. proposed that implementing DL solves autofocusing in digital holography by correctly labelling a hologram or predicting the accurate distance. The trained CNN could predict distance without the knowledge of parameters such as exposure time, object, or incident angle. The time consumed was observed to be lesser compared to that of conventional autofocussing techniques (Ren et al. 2018). Gupta et al. demonstrated that CNN when combined with DHM achieved 91.3% accuracy in the classification of immune cells with a high throughput rate of more than 100 cells per second. This proved to be an automated and robust method for application in clinical studies (Gupta et al. 2019). Conventional digital holography imaging (DHI) algorithms used many iterations to recover reconstructed focussed images and they were found to be time-consuming. Zhang et al. reported that a U-net CNN could be implemented to recover the original phase of the sample to a great extent. This was done by producing data sets to simulate various degrees of defocused image. This approach was applied in a real-time off-axis digital holographic microscope and was found to be feasible, accurate, and fast. To improve image quality, future research may concentrate on the removal of complex background additional phases (Zhang et al. 2018a, b).

**Fig. 6:**
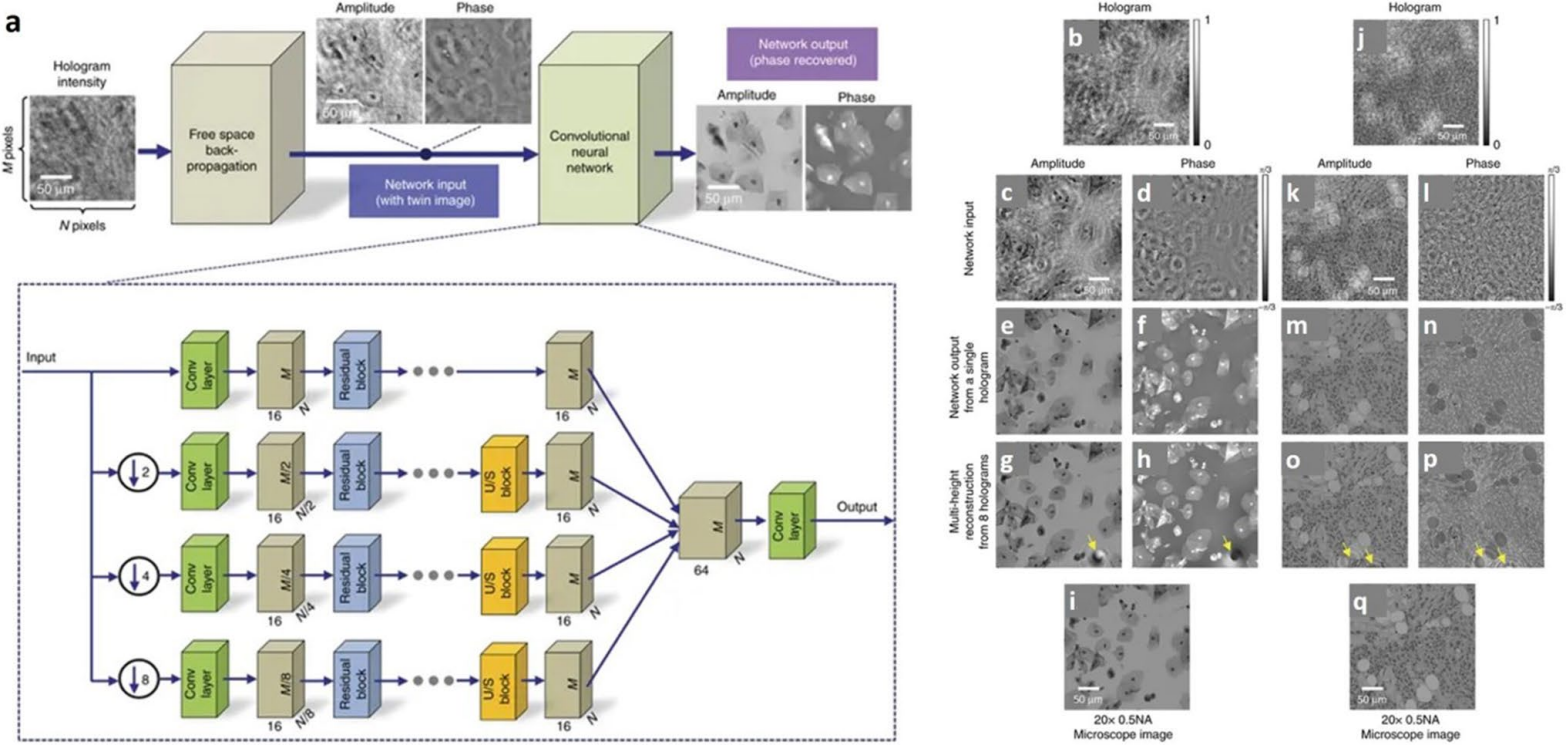
**a** Architecture of deep neural network is composed of convolutional layers, residual blocks, and upsampling blocks which blindly outputs artefact-free phase and amplitude images of the object using only one hologram intensity. Comparison of the holographic reconstruction results for different types of samples: **b**–**i** Pap smear, **j**–**q** breast tissue section. **b**, **j** Zoomed-in regions of interest from the acquired holograms. **c**, **d**, **k**, **l** Amplitude and phase images resulting from free-space back-propagation of a single hologram intensity, shown in **b** and **j**, respectively. **e**, **f**, **m**, **n** Corresponding amplitude and phase images of the same samples obtained by the deep neural network, demonstrating the blind recovery of the complex object image without twin-image and self-interference artefacts using a single hologram. **g**, **h**, **o**, **p** Amplitude and phase images of the same samples were reconstructed using multi-height phase retrieval with 8 holograms acquired at different sample-to-sensor distances. **q** Corresponding bright-field microscopy images of the same samples, shown for comparison. The yellow arrows point to artifacts in **g**, **h**, **o**, **p** (due to out-of-focus dust particles or other unwanted objects) that are significantly suppressed by the network reconstruction, as shown in **e**, **f**, **m**, **n**. This figure is adapted with permission from Rivenson et al. 2018a, Springer Nature

In-line holography systems require phase recovery as an essential component which requires reconstructing multiple intensity holographic images/holograms. Rivenson et al. showed that a CNN-based rapid method could be used for phase recovery and image reconstruction by a single intensity hologram. A graphic processing unit (GPU)-based laptop requires 3.11 s to recover phase and sample amplitude images for a FOV of 1 mm^2^. It was observed that this process was able to remove twin images and artefacts related to self-interference. The method was validated for Pap and blood smears. The CNN-based method used for this study, predominantly processed images by eliminating defocussed interference artefacts that may appear due to dust particles present on different surfaces of the imaging arrangement (Fig. 6) (Rivenson et al. 2018a). Digital holographic microscopy allows the 3D reconstruction of samples throughout the volume from a snapshot of a single hologram, but the quality of reconstructions may be compromised by fringe interference. Wu et al. implemented an effective method combining the contrast of bright field microscopy with holographic image reconstruction using GAN. Snapshot imaging of bioaerosols in 3D was found to match the contrast and axial sectioning of a high numerical aperture bright-field microscope. This cross-modality method eliminated the need to scan the volumetric sample (Wu et al. 2019a, b). Wu et al. reported that a CNN-based method was used to increase DOF by performing autofocusing and phase recovery. The deep-learning-based framework known as holographic imaging using deep Learning for Extended Focus (HIDEF), used random pairs of defocussed holograms and focussed phase-recovered images. This resulted in decreased time complexity of image reconstruction in 3D by simultaneously refocussing and phase recovery (Wu et al. 2018).

Pitkäaho et al. demonstrated the determination of focussed reconstruction depth of Madin-Darby canine kidney cell clusters using DL. CNN was trained with focussed depths of many holograms, and it later predicted the depth with high accuracy without any numerical propagation (Pitkäaho et al. 2019). Zhang et al. proposed a deep phase shifter that was used to produce multiple interferograms from a single interferogram. It was reported that a deep-phase-shift network (DPS-net) was trained with simulation data set and it could be used in phase recovery for real samples. It was found to be a feasible, precise method, with the potential to improve the quantitative phase imaging technology (Zhang et al. 2020a, b, c). It has been observed that for the early diagnosis of parasitic infection, usually, the present screening techniques struggle to provide adequate sensitivity and volumetric throughput. Zhang et al. demonstrated a label-free motility-based computational imaging device to detect motile parasites in the body fluids. Detection of the parasites in a bodily fluid is tedious due to the presence of a large number of red blood cells, white blood cells and platelets in comparison with a limited number of parasites (100 parasites/mL) in the sample. Also, state-of-the-art immunology techniques such as polymerase chain reaction (PCR) and rapid diagnostic tests (RDTs) are limited by sensitivity. Hence, the holographic speckle analysis algorithm with deep learning could be the best method that can be used to detect trypanosomes in the whole blood and cerebrospinal fluid. It was reported that 10 trypanosomes per mL of blood and three trypanosomes per mL of CSF were detected. The method was found to be fast, accurate, and cost-effective for the detection of parasites (Zhang et al. 2018a, b). Lens-free holographic microscopy (LFHM) is a profitable instrument for large FOV imaging. Lou et al. proposed a DL-based pixel super-resolution (PSR) method that overcomes the resolution limit of LFHM with the help of a sequence of sub-pixel shifted lesser resolution (LR) lens-free holograms to create a high-resolution hologram (HR). This approach could notably increase the acquisition of data and the high-resolution hologram reconstruction processes, hence providing a solution to lens-free, high-resolution imaging. This method successfully overcame the resolution limit of LFHM. With the growth in DL-based holographic imaging and the computing power of GPU, fast and large FOV super-resolution lens-free imaging for screening and lab-on-a-chip applications be achieved (Luo et al. 2019).

Deep neural networks (DNNs) have been extensively used for QPI, especially for transparent samples. An untrained DNN, which does not need training data was used for measurement, imaging, and phase reconstruction (Ye et al. 2020). Ye et al. demonstrated the latest phase retrieval approach on DNN. The approach has simplified the procedure of measuring by giving one interferogram as input and QPM based on off-axis holography measures it and gives QPI as an output image. It has made real-time phase retrieval easier by abolishing the need for phase unwrapping. The method was found to be similar when compared with the Fourier Transform method of phase retrieval. DNN-based phase retrieval may result in wider application of QPM in material metrology and bioimaging in the future (Bostan et al. 2020). A DL-based network, conditional GAN (C-GAN) can remove twin-image noise from the phase images of Gabor holography which were trained by a quantitative contrast-phase image from the off-axis digital holography. A human red blood cell and elliptical cancer models were trained, and the biochemical properties were quantified. The model was able to recover other elliptical cell lines as well. The misalignments could be neglected especially in the case of incorrect reconstruction distance as this model could still extract in-focus images (Moon et al. 2020). O’Connor et al. demonstrated a first DL approach in the identification of cells and diagnosis of disease with the help of spatio-temporal information of cells retrieved by a DHM system. Shearing DHM could record live cells and phase profiles were reconstructed for the classification at each time instances of segmented cells. This data was then input in the recurrent bi-directional long short-term memory (Bi-LSTM) network which in turn classify cells depending on their time-varying behavior. The proposed approach showed better performance compared to traditional machine learning methods on a dataset of diseased and healthy human red blood cells (O’Connor et al. 2020). Based on the morphology and motility observed by Bright field microscopy, a sperm cell is selected for the intracytoplasmic sperm injection (ICSI) procedure of assisted reproductive technology (ART). However, to identify and distinguish even the smallest morphological feature which may affect the fertilizing potential of the sperm cell, bright field contrast is not sufficient. A partially spatially coherent DHM (PSC-DHM) was developed to distinguish between normal sperm cells and the ones under stress conditions like cryopreservation, around hydrogen peroxide and ethanol. Using the data from PSC-DHM, phase maps for normal and stress-conditioned sperm cells were reconstructed and classified by DNN. During the validation using the test dataset, specificity of 84.88%, the sensitivity of 95.03% and accuracy of 85% were obtained. DNN-based QPI can be further applied for diagnosing and classifying semen based on its quality and fertilizing ability (Butola et al. 2020).

### Raman microscopy

Raman spectroscopy is one of these techniques that have managed to gain considerable attention, as it is non-destructive and provides precise information at a molecular level (Mahadevan-Jansen and Richards-Kortum 1996; Stone et al. 2004). It is an efficient analytical technique that helps to study the structure and binding of molecules by examining their scattering properties. The sensitivity of Raman spectrometers has helped to obtain high-quality Raman spectra from tissues and cells. Confocal Raman microscopy was used for the detection of lung cancer. The stained sections of normal lung tissue were used as a reference and scanned with a microscope. Mean spectra are calculated from these, and two spectra were obtained for malignant and normal tissues. A data reduction technique called principal component analysis (PCA) has been used in combination with Raman microscopy to analyze the presence of variation among the spectra by reducing it to a smaller number of principal components. PCA, along with Raman microscopy, has therefore been applied to distinguish normal and malignant lung tissue with a sensitivity and specificity of 84% and 61%, respectively. Many subtle differences in intensities between the two main spectra can be identified along with the main differences in intensity of the Amide I band in lung tumors. Raman microscopy of tissue sections can differentiate between malignant and normal bronchial tissue and predict the postoperative occurrence of cancer (Magee et al. 2010). Although, the spontaneous Raman technique is used for various biomedical applications, however, it suffers from small scattering cross-section, sensitivity and live-cell imaging.

**Fig. 7:**
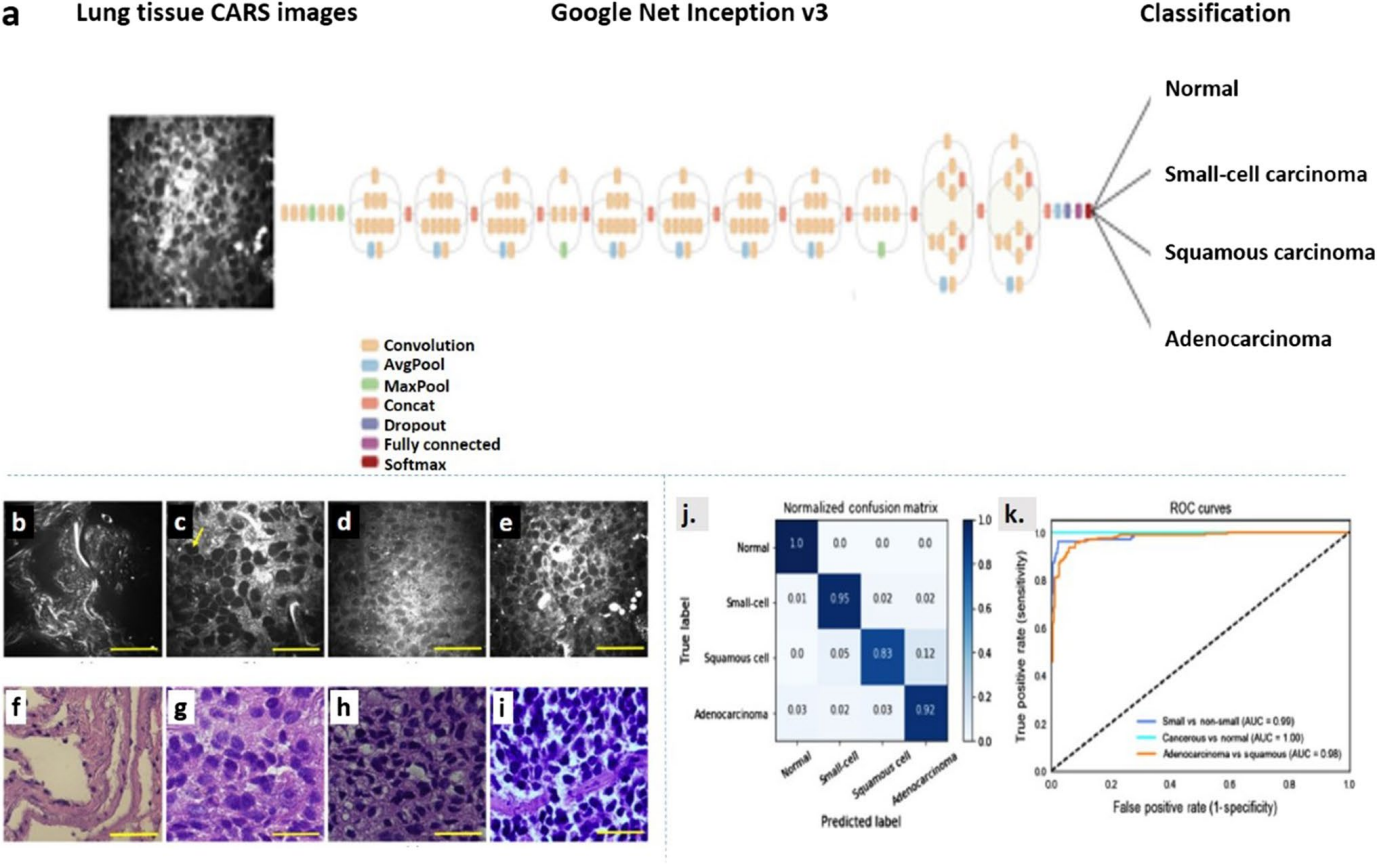
Coronal mouse brain SRS images acquired at 2990 cm^−1^. **a** Low-power image acquired at 1 mW Stokes and 20 mW pump. **b** The low-power image denoised with VST. **c** The low-power image is denoised with the deep learning algorithm. **d** The high power image acquired at 20 mW Stokes and 20 mW pump. Two-color (lipids and proteins) composite SRS images of ex vivo mouse brain and corresponding pixel value-line plots. **e** Low-power image acquired at 1 mW Stokes 20 mW pump. **f** The low-power image is denoised using VST. **g** The low-power image is denoised with the deep learning algorithm. **h** The high-power image acquired at 20 mW Stokes and pump. Pixel value-line plots along the red line are shown for each composite image; Two-color (lipids-green, proteins-blue) SRS images of a coronal mouse-brain slice. This figure is adapted with permission from Manifold et al. 2017, SPIE

Stimulated Raman scattering (SRS) microscopy is a quantitative label-free chemical imaging method that has exhibited biomedical imaging utilities. However, the absorption and scattering of light often cause a low signal-to-noise ratio (SNR) and low image quality. Manifold et al. proposed the application of a deep learning algorithm to denoise the signal and to retain the quality of the image at the same time. The DL algorithm was based on U-net CNN, which was used for the images at different imaging depths, power, and zoom. Hence, deep learning was found to be a powerful tool in imaging, where parameters were not constant and ground-truth images were not used to create a supervised learning training set. Other denoising algorithms used, resulted in blurred biological features of the image (Fig. 7) (Manifold et al. 2019). A study by Suzuki et al. reported the use of CNN structure (VGG-16) in the analysis of images of blood cells and *E. gracilis* obtained from the SRS imaging flow cytometer by classifying different cell types (Suzuki et al. 2019). This technique is label-free and hence it overcomes the cytotoxicity problem faced with conventional flow cytometry. Further, it showed a high throughput of 140 cells/s. Another application was found in cancer diagnosis. Acquiring a 3-D assessment of tumor edges was difficult and the presence of sub-mucosal extension may cause recurrence of the tumor. Hence, Zhang et al. demonstrated that a deep learning model, ResNet combined with SRS imaging has the potential for intraoperative diagnosis of such resection margins. This model may be further optimized for robust and automated prediction in the future (Zhang et al. 2019).

**Fig. 8:**
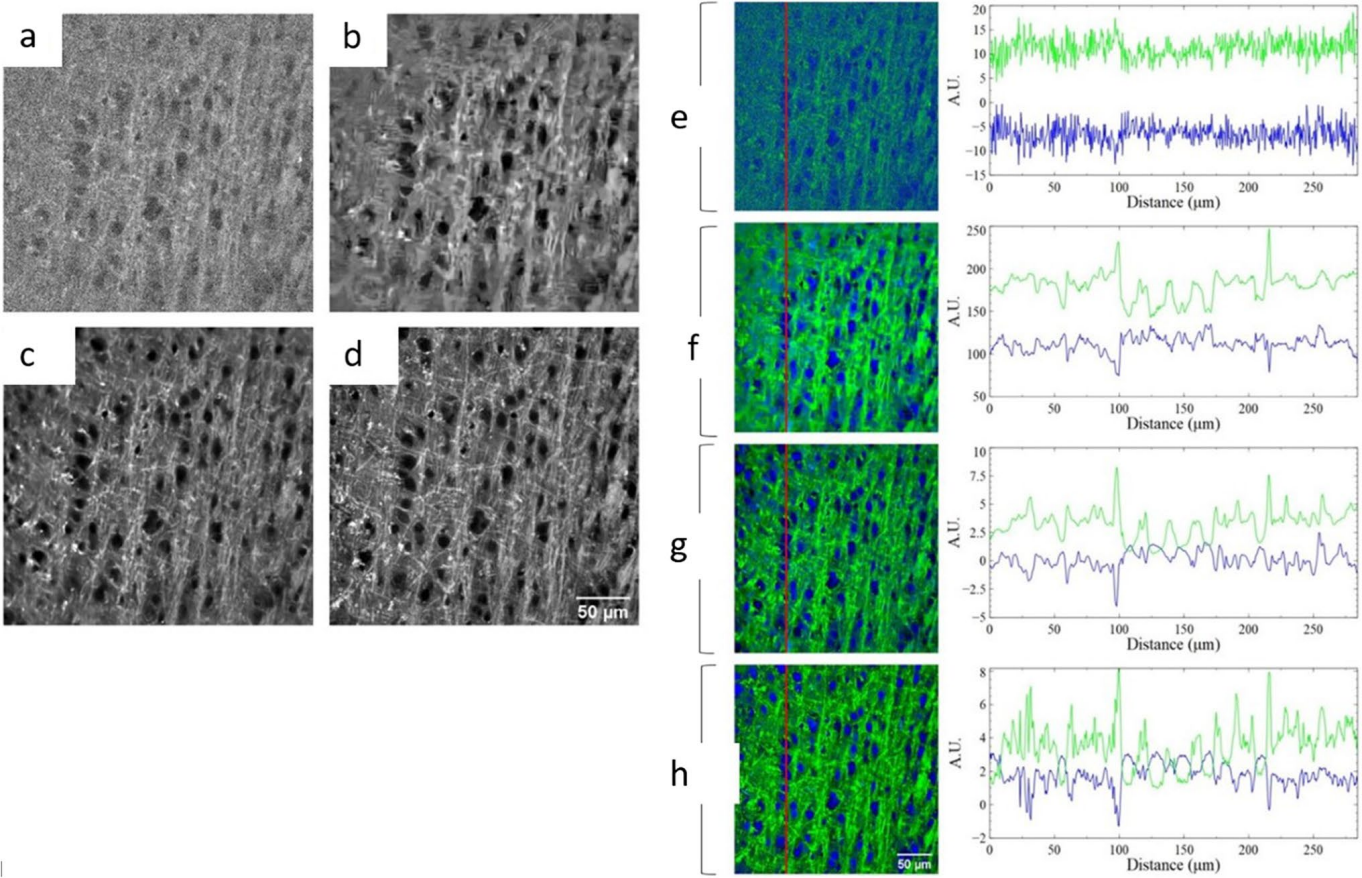
**a** Transfer learning layout. A GoogleNet Inception v3 CNN, pre-trained on the ImageNet data is fine-tuned with CARS images comprising four classes: normal, small-cell carcinoma, squamous carcinoma, and adenocarcinoma. **b**–**e** Representative CARS images and **f**–**i** corresponding H&E-stained images of human lung tissues: **b**, **f** normal lung, **c**, **g** adenocarcinoma, **d**, **h** squamous cell carcinoma, and **e**, **i** small-cell carcinoma. Scale bars: 50 μm. Deep CNN model performance on the **j** normalized confusion matrix. Each row represents the instances in a ground-truth class and the value in each column represents what percentage of the images is predicted to a certain class. **k** ROC curves for three conditions: separating cancerous from normal lung images (light blue); separating small-cell carcinoma from nonsmall cell carcinoma lung images (dark blue); separating adenocarcinoma and squamous carcinoma lung images (orange). AUC scores are given in the legend. This figure is adapted with permission from Weng et al. 2017, SPIE

CARS is a label-free imaging technique with great potential in cancer diagnosis. However, conventional methods face challenges due to low signal-to-noise ratio and uneven background. They also perform inefficient CARS image segmentation. Various studies have shown that the DL-based CARS image analysis has been able to overcome these challenges with effective and automated nuclei segmentation. Hammoudi et al. demonstrated a fully automated approach for nuclei segmentation by combining superpixels and artificial neural networks for nuclei identification in CARS images of lungs. The nuclei segmentation was performed without even looking for cell morphology which was a very critical step. Results proved that this approach showed great potential in nuclei segmentation (Hammoudi et al. 2011). In a study by Weng et al. the application of DL algorithms to CARS images was shown for cancer tissue identification in the lungs. A CNN was pre-trained on ImageNet data and retrained with CARS images using the transfer learning method (illustrated in Fig. 8). This method led to a reduced processing time with real-time analysis. Additionally, this method could also differentiate between cancerous lung tissue and normal lung tissue with an accuracy of 89.2%. With respect to the current research and interesting developments, a miniaturized CARS imaging method for fiber-based micro-endoscopic imaging and a multimodal image classification algorithm for real-time tissue detection would be desirable in the future (Weng et al. 2017).

## Limitations of DL

Despite the many advantages of DL algorithms for the accurate assessment of microscopic images, there are a few limitations. Biophotonics is a growing field, and the availability of an all-inclusive clinical dataset is critical DL requires a large amount of annotated data to perform an underlying analysis (Dumur et al. 2019). Using a small dataset to train the DL models could lead to overfitting that cause poor generalizability of the dataset. To reduce the need for supervised learning methods, approaches such as transfer learning, unsupervised learning, and weakly supervised learning could be adopted. In transfer learning a trained algorithm is restructured and used according to the requirement whereas in weekly supervised/unsupervised methods the model is trained with the raw data. However, they have not been proven as effective as supervised learning (Hägele et al. 2020; Zhang et al. 2020a, b, c). Moreover, DL performs better on images from benchmarked datasets as compared to images from an alien dataset, calling for time-consuming training (Hesamian et al. 2019). Along with this, the lack of variability in the training dataset causes an imbalance and greatly affects the performance of the model. Additionally, the direct implementation of DL models in the healthcare system is a crucial issue, since the performance of the model is still not known.

## Conclusion

The applications of DL-based image processing methods in optical microscopy have been found to improve the image quality and spatial resolution with automated analysis of the acquired images (Rivenson et al. 2017; Caicedo et al. 2019). In recent years, the availability of an enormous amount of data and high-speed computing systems has led to the rapid development of DL techniques. By using publicly available data sets it is possible to develop algorithms from scratch and it is also feasible to use pre-trained networks such as GoogleNet, Vgg-16, and UNet, with transfer learning (Mohanty et al. 2016). Such techniques have proven to enhance image resolution in various microscopic techniques and enable automated disease classification (Li et al. 2018). Additionally, DL in optical microscopy is also used to monitor gene expression and protein localization in organisms (Silvestri et al. 2015). The application of DL in smartphone-based microscopy has led to the development of portable devices which facilitate the early detection of cancer (Rivenson et al. 2018b; Wei et al. 2014; Uthoff et al. 2018; Bornhorst et al. 2019). Holographic image reconstruction has been made easy with the use of DL, which helps in the accurate reconstruction of the image with reduced hardware requirement (Jo et al. 2017; Rivenson et al. 2018a). Accordingly, CNN-based DL networks employed in medical image processing have emerged as one of the potential tools in image enhancement and prediction.

## References

[CR1] Bai C, Liu C, Yu X, Peng T, Min J, Yan S, Dan D, Yao B (2019). Imaging enhancement of light-sheet fluorescence microscopy via deep learning. IEEE Photonics Technol Lett.

[CR2] Belthangady C, Royer LA (2019). Applications, promises, and pitfalls of deep learning for fluorescence image reconstruction. Nat Methods.

[CR3] Bornhorst J, Nustede EJ, Fudickar S (2019). Mass surveillance of C. elegans-smartphone-based DIY microscope and machine-learning-based approach for worm detection. Sensors.

[CR4] Bostan E, Heckel R, Chen M, Kellman M, Waller L (2020). Deep phase decoder: self-calibrating phase microscopy with an untrained deep neural network. Optica.

[CR5] Butola A, Popova D, Prasad DK, Ahmad A, Habib A, Tinguely JC, Basnet P, Acharya G, Senthilkumaran P, Mehta DS, Ahluwalia BS (2020). High spatially sensitive quantitative phase imaging assisted with deep neural network for classification of human spermatozoa under stressed condition. Sci Rep.

[CR6] Caicedo JC, Roth J, Goodman A, Becker T, Karhohs KW, Broisin M, Molnar C, McQuin C, Singh S, Theis FJ, Carpenter AE (2019). Evaluation of deep learning strategies for nucleus segmentation in fluorescence images. Cytometry A.

[CR7] Chen T, Chefd’hotel C (2014) Deep learning based automatic immune cell detection for immunohistochemistry images. In International workshop on machine learning in medical imaging: 17-2410.1007/978-3-319-10581-9_3

[CR8] Chen Y, Lin Z, Zhao X, Wang G, Gu Y (2014). Deep learning-based classification of hyperspectral data. IEEE J Sel to Appl Earth Obs Remote Sens.

[CR9] Corsetti S, Wijesinghe P, Poulton PB, Sakata S, Vyas K, Herrington CS, Nylk J, Gasparoli F, Dholakia K (2020). Widefield light sheet microscopy using an Airy beam combined with deep-learning super-resolution. OSA Continuum.

[CR10] Deng L, Liu Y (2018) Deep learning in natural language processing, First ed. Springer

[CR11] Dumur T, Duncan S, Graumann K, Desset S, Randall RS, Scheid OM, Bass HW, Prodanov D, Tatout C, Baroux C (2019). Probing the 3D architecture of the plant nucleus with microscopy approaches: challenges and solutions. Nucleus.

[CR12] Elman JL (1990). Finding structure in time. Cogn Sci.

[CR13] Esteva A, Kuprel B, Novoa RA, Ko J, Swetter SM, Blau HM, Thrun S (2017). Dermatologist-level classification of skin cancer with deep neural networks. Nature.

[CR14] Gallardo-Caballero R, García-Orellana CJ, García-Manso A, González-Velasco HM, Tormo-Molina R, Macías-Macías M (2019). Precise pollen grain detection in bright field microscopy using deep learning techniques. Sensors.

[CR15] Gupta RK, Chen M, Malcolm GP, Hempler N, Dholakia K, Powis SJ (2019). Label-free optical hemogram of granulocytes enhanced by artificial neural networks. Opt Express.

[CR16] Hägele M, Seegerer P, Lapuschkin S, Bockmayr M, Samek W, Klauschen F, Müller KR, Binder A (2020). Resolving challenges in deep learning-based analyses of histopathological images using explanation methods. Sci Rep.

[CR17] Hammoudi AA, Li F, Gao L, Wang Z, Thrall MJ, Massoud Y, Wong ST (2011) Automated nuclear segmentation of coherent anti-Stokes Raman scattering microscopy images by coupling superpixel context information with artificial neural networks. Int Workshop Machine Learning in Medical Imaging, 317-32510.1007/978-3-642-24319-6_39

[CR18] Hay EA, Parthasarathy R (2018). Performance of convolutional neural networks for identification of bacteria in 3D microscopy datasets. PLOS Comput Biol.

[CR19] Hesamian MH, Jia W, He X, Kennedy P (2019). Deep learning techniques for medical image segmentation: achievements and challenges. J Digit Imaging.

[CR20] Hornik K, Stinchcombe M, White H (1989). Multilayer feedforward networks are universal approximators. Neural Netw.

[CR21] Hutter F, Kotthoff L, Vanschoren J (2019) Automated machine learning: methods, systems, challenges, Springer Nature

[CR22] Huttunen MJ, Hassan A, McCloskey CW, Fasih S, Upham J, Vanderhyden BC, Boyd RW, Murugkar S (2018). Automated classification of multiphoton microscopy images of ovarian tissue using deep learning. J Biomed Opt.

[CR23] Izadyyazdanabadi M, Belykh E, Zhao X, Moreira LB, Gandhi S, Cavallo C, Eschbacher J, Nakaji P, Preul MC, Yang Y (2019). Fluorescence image histology pattern transformation using image style transfer. Front Oncol.

[CR24] Jiao Y, Schneider BS, Regentova E, Yang M (2019). DeepQuantify: deep learning and quantification system of white blood cells in light microscopy images of injured skeletal muscles. J Med Imaging.

[CR25] Jo JY, Park S, Jung JH, Yoon J, Joo H, Kim MH, Kang SJ, Choi MC, Lee SY, Park Y (2017). Holographic deep learning for rapid optical screening of anthrax spores. Sci Adv.

[CR26] Kim G, Jo Y, Cho H, Min HS, Park Y et al (2019) Learning-based screening of hematologic disorders using quantitative phase imaging of individual red blood cells. Biosen Bioelectron 123:69–76. 10.1016/j.bios.2018.09.068 10.1016/j.bios.2018.09.068 30321758

[CR27] Kobayashi H, Lei C, Wu Y, Mao A, Jiang Y, Guo B, Ozeki Y, Goda K (2017). Label-free detection of cellular drug responses by high-throughput bright-field imaging and machine learning. Sci Rep.

[CR28] Kraus OZ, Grys BT, Ba J, Chong Y, Frey BJ, Boone C, Andrews BJ (2017). Automated analysis of high-content microscopy data with deep learning. Mol Syst Biol.

[CR29] Krueger R, Beyer J, Jang WD, Kim NW, Sokolov A, Sorger PK, Pfister H (2019). Facetto: combining unsupervised and supervised learning for hierarchical phenotype analysis in multi-channel image data. IEEE Trans vis Comput Graph.

[CR30] LeCun Y, Bengio Y, Hinton G (2015). Deep learning. Nature.

[CR31] Li Y, Xu F, Zhang F, Xu P, Zhang M, Fan M, Li L, Gao X, Han R (2018). DLBI: deep learning guided Bayesian inference for structure reconstruction of super-resolution fluorescence microscopy. Bioinformatics.

[CR32] Li Y, Zheng R, Wu Y, Chu K, Xu Q, Sun M, Smith ZJ (2019). A low-cost, automated parasite diagnostic system via a portable, robotic microscope and deep learning. J Biophotonics.

[CR33] Litjens G, Sánchez CI, Timofeeva N, Hermsen M, Nagtegaal I, Kovacs I, Hulsbergen-Van De Kaa C, Bult P, Van Ginneken B, Van Der Laak J (2016). Deep learning as a tool for increased accuracy and efficiency of histopathological diagnosis. Sci Rep.

[CR34] Liu J, Huang X, Chen L, Tan S (2020). Deep learning-enhanced fluorescence microscopy via degeneration decoupling. Opt Express.

[CR35] Liu S, Nie J, Li Y, Yu T, Zhu D, Fei P (2017). Three-dimensional, isotropic imaging of mouse brain using multi-view deconvolution light sheet microscopy. J Innov Opt Health Sci.

[CR36] Luo Z, Yurt A, Stahl R, Lambrechts A, Reumers V, Braeken D, Lagae L (2019). Pixel super-resolution for lens-free holographic microscopy using deep learning neural networks. Opt Express.

[CR37] Magee ND, Beattie JR, Carland C, Davis R, McManus K, Bradbury I, Fennell DA, Hamilton P, Ennis M, McGarvey JJ, Elborn JS (2010). Raman microscopy in the diagnosis and prognosis of surgically resected nonsmall cell lung cancer. J Biomed Opt.

[CR38] Mahadevan-Jansen A, Richards-Kortum R (1996). Raman spectroscopy for the detection of cancers and precancers. J Biomed Opt.

[CR39] Malkiel I, Mrejen M, Nagler A, Arieli U, Wolf L, Suchowski H (2018). Plasmonic nanostructure design and characterization via deep learning. Light Sci Appl.

[CR40] Manifold B, Thomas E, Francis AT, Hill AH, Fu D (2019). Denoising of stimulated Raman scattering microscopy images via deep learning. Biomed Opt Express.

[CR41] Mazumder N, Qiu J, Kao FJ, Diaspro A (2017). Mueller matrix signature in advanced fluorescence microscopy imaging. J Optics.

[CR42] Mohanty SP, Hughes DP, Salathé M (2016). Using deep learning for image-based plant disease detection. Front Plant Sci.

[CR43] Moon I, Jaferzadeh K, Kim Y, Javidi B (2020). Noise-free quantitative phase imaging in Gabor holography with conditional generative adversarial network. Opt Express.

[CR44] Nielsen MA (2015). Neural networks and deep learning.

[CR45] O’Connor T, Anand A, Andemariam B, Javidi B (2020). Deep learning-based cell identification and disease diagnosis using spatio-temporal cellular dynamics in compact digital holographic microscopy. Biomed Opt Express.

[CR46] Ounkomol C, Seshamani S, Maleckar MM, Collman F, Johnson GR (2018). Label-free prediction of three-dimensional fluorescence images from transmitted-light microscopy. Nat Methods.

[CR47] Pinkard H, Phillips Z, Babakhani A, Fletcher DA, Waller L (2019). Deep learning for single shot auto-focus microscopy. Optica.

[CR48] Pitkäaho T, Manninen A, Naughton TJ (2019). Focus prediction in digital holographic microscopy using deep convolutional neural networks. Appl Opt.

[CR49] Pradhan P, Guo S, Ryabchykov O, Popp J, Bocklitz TW (2020). Deep learning a boon for biophotonics?. J Biophotonics.

[CR50] Rahman TY, Mahanta LB, Chakraborty C, Das AK, Sarma JD (2018). Textural pattern classification for oral squamous cell carcinoma. J Microsc.

[CR51] Rehman A, Abbas N, Saba T, Rahman SI, Mehmood Z, Kolivand H (2018). Classification of acute lymphoblastic leukemia using deep learning. Microsc Res Tech.

[CR52] Ren Z, Xu Z, Lam EY (2018) Autofocusing in digital holography using deep learning. In Three-dimensional and multidimensional microscopy: image acquisition and processing XXV 104991V10.1117/12.2289282

[CR53] Rieckher M, Kyparissidis-Kokkinidis I, Zacharopoulos A, Kourmoulakis G, Tavernarakis N, Ripoll J, Zacharakis G (2015). A customized light sheet microscope to measure spatio-temporal protein dynamics in small model organisms. PLoS ONE.

[CR54] Rivenson Y, Göröcs Z, Günaydin H, Zhang Y, Wang H, Ozcan A (2017). Deep Learning Microscopy Optica.

[CR55] Rivenson Y, Zhang Y, Günaydın H, Teng D, Ozcan A (2018). Phase recovery and holographic image reconstruction using deep learning in neural networks. Light Sci Appl.

[CR56] Rivenson Y, CeylanKoydemir H, Wang H, Wei Z, Ren Z, Günaydın H, Zhang Y, Gorocs Z, Liang K, Tseng D, Ozcan A (2018). Deep learning enhanced mobile-phone microscopy. ACS Photonics.

[CR57] Rivenson Y, Liu T, Wei Z, Zhang Y, de Haan K, Ozcan A (2019). PhaseStain: the digital staining of label-free quantitative phase microscopy images using deep learning. Light Sci Appl.

[CR58] Rivenson Y, Wu Y, Ozcan A (2019). Deep learning in holography and coherent imaging. Light Sci Appl.

[CR59] Silvestri L, Paciscopi M, Soda P, Biamonte F, Iannello G, Frasconi P, Pavone FS (2015). Quantitative neuroanatomy of all Purkinje cells with light sheet microscopy and high-throughput image analysis. Front Neuroanat.

[CR60] Sozaki A, Mikami H, Hiramatsu K, Sakuma S, Kasai Y, Iino T, Yamano T, Yasumoto A, Oguchi Y, Suzuki N, Shirasaki YA (2019). A practical guide to intelligent image-activated cell sorting. Nat Protoc.

[CR61] Stone N, Kendall C, Smith J, Crow P, Barr H (2004). Raman spectroscopy for identification of epithelial cancers. Faraday Discuss.

[CR62] Suzuki Y, Kobayashi K, Wakisaka Y, Deng D, Tanaka S, Huang CJ, Lei C, Sun CW, Liu H, Fujiwaki Y, Lee S (2019). Label-free chemical imaging flow cytometry by high-speed multicolour stimulated Raman scattering. PNAS.

[CR63] Thierbach K, Bazin PL, Gavriilidis F, Kirilina E, Jäger C, Morawski M, Geyer S, Weiskopf N, Scherf N (2018) Deep learning meets topology-preserving active contours: towards scalable quantitative histology of cortical cytoarchitecture. bioRxiv 297689. 10.1101/297689

[CR64] Uthoff RD, Song B, Sunny S, Patrick S, Suresh A, Kolur T, Keerthi G, Spires O, Anbarani A, Wilder-Smith P, Kuriakose MA (2018). Point-of-care, smartphone-based, dual-modality, dual-view, oral cancer screening device with neural network classification for low-resource communities. PLoS ONE.

[CR65] Wagner N, Beuttenmueller F, Norlin N, Gierten J, Boffi JC, Wittbrodt J, Weigert M, Hufnagel L, Prevedel R, Kreshuk A (2021). Deep learning-enhanced light-field imaging with continuous validation. Nat Methods.

[CR66] Wang H, Rivenson Y, Jin Y, Wei Z, Gao R, Günaydın H, Bentolila LA, Kural C, Ozcan A (2019). Deep learning enables cross-modality super-resolution in fluorescence microscopy. Nat Methods.

[CR67] Wang X, Jiang W, Luo Z (2016) Combination of convolutional and recurrent neural network for sentiment analysis of short texts. Proceedings of COLING 2016, the 26th international conference on computational linguistics: Technical papers 2428–2437

[CR68] Wei Q, Luo W, Chiang S, Kappel T, Mejia C, Tseng D, Chan RY, Yan E, Qi H, Shabbir F, Ozkan H (2014). Imaging and sizing of single DNA molecules on a mobile phone. ACS Nano.

[CR69] Weigert M, Schmidt U, Boothe T, Müller A, Dibrov A, Jain A, Wilhelm B, Schmidt D, Broaddus C, Culley S, Rocha-Martins M (2019). Content-aware image restoration: pushing the limits of fluorescence microscopy. Nat Methods.

[CR70] Weng S, Xu X, Li J, Wong ST (2017). Combining deep learning and coherent anti-Stokes Raman scattering imaging for automated differential diagnosis of lung cancer. J Biomed Opt.

[CR71] Wu YC, Shiledar A, Li YC, Wong J, Feng S, Chen X, Chen C, Jin K, Janamian S, Yang Z, Ballard ZS (2017). Air quality monitoring using mobile microscopy and machine learning. Light Sci Appl.

[CR72] Wu Y, Rivenson Y, Zhang Y, Wei Z, Günaydin H, Lin X, Ozcan A (2018). Extended depth-of-field in holographic imaging using deep-learning-based autofocusing and phase recovery. Optica.

[CR73] Wu Y, Luo Y, Chaudhari G, Rivenson Y, Calis A, De Haan K, Ozcan A (2019). Bright-field holography: cross-modality deep learning enables snapshot 3D imaging with bright-field contrast using a single hologram. Light Sci Appl.

[CR74] Wu Y, Rivenson Y, Wang H, Luo Y, Ben-David E, Bentolila LA, Pritz C, Ozcan A (2019). Three-dimensional virtual refocusing of fluorescence microscopy images using deep learning. Nat Methods.

[CR75] Xiao L, Fang C, Zhu L, Wang Y, Yu T, Zhao Y, Zhu D, Fei P (2020). Deep learning-enabled efficient image restoration for 3D microscopy of turbid biological specimens. Opt Express.

[CR76] Yang SJ, Berndl M, Ando DM, Barch M, Narayanaswamy A, Christiansen E, Hoyer S, Roat C, Hung J, Rueden CT, Shankar A (2018). Assessing microscope image focus quality with deep learning. BMC Bioinformatics.

[CR77] Yao R, Ochoa M, Yan P, Intes X (2019). Net-FLICS: fast quantitative wide-field fluorescence lifetime imaging with compressed sensing – a deep learning approach. Light Sci Appl.

[CR78] Ye Y, Shu X, Zhou R (2020). Deep learning based phase retrieval in quantitative phase microscopy. In Unconventional Optical Imaging.

[CR79] Zhang G, Guan T, Shen Z, Wang X, Hu T, Wang D, He Y, Xie N (2018). Fast phase retrieval in off-axis digital holographic microscopy through deep learning. Opt Express.

[CR80] Zhang J (2017). Multivariate analysis and machine learning in cerebral palsy research. Front Neurol.

[CR81] Zhang L, Wu Y, Zheng B, Su L, Chen Y, Ma S, Hu Q, Zou X, Yao L, Yang Y, Chen L (2019). Rapid histology of laryngeal squamous cell carcinoma with deep learning based stimulated Raman scattering microscopy. Theranostics.

[CR82] Zhang Q, Liu Y, Gong C, Chen Y, Yu H (2020). Applications of deep learning for dense scenes analysis in agriculture: a review. Sensors.

[CR83] Zhang Q, Lu S, Li J, Li W, Li D, Lu X, Zhong L, Tian J (2020b) Deep phase shifter for quantitative phase imaging. arXiv preprint 03027

[CR84] Zhang Y, Koydemir HC, Shimogawa MM, Yalcin S, Guziak A, Liu T, Oguz I, Huang Y, Bai B, Luo Y, Luo Y (2018). Motility-based label-free detection of parasites in bodily fluids using holographic speckle analysis and deep learning. Light Sci Appl.

[CR85] Zhang Y, Xie Y, Liu W, Deng W, Peng D, Wang C, Xu H, Ruan C, Deng Y, Guo Y, Lu C (2020). DeepPhagy: a deep learning framework for quantitatively measuring autophagy activity in Saccharomyces cerevisiae. Autophagy.

